# Multiple case study of processes used by hospitals to select performance indicators: do they align with best practices?

**DOI:** 10.1093/intqhc/mzae011

**Published:** 2024-02-19

**Authors:** Michael A Heenan, Glen E Randall, Jenna M Evans, Erin M Reid

**Affiliations:** DeGroote School of Business, McMaster University, Hamilton, Ontario L8S 4M4, Canada; DeGroote School of Business, McMaster University, Hamilton, Ontario L8S 4M4, Canada; DeGroote School of Business, McMaster University, Hamilton, Ontario L8S 4M4, Canada; DeGroote School of Business, McMaster University, Hamilton, Ontario L8S 4M4, Canada

**Keywords:** quality, performance, measurement, engagement

## Abstract

Several health policy institutes recommend reducing the number of indicators monitored by hospitals to better focus on indicators most relevant to local contexts. To determine which indicators are the most appropriate to eliminate, one must understand how indicator selection processes are undertaken. This study classifies hospital indicator selection processes and analyzes how they align with practices outlined in the 5-P Indicator Selection Process Framework. This qualitative, multiple case study examined indicator selection processes used by four large acute care hospitals in Ontario, Canada. Data were collected through 13 semistructured interviews and document analysis. A thematic analysis compared processes to the 5-P Indicator Selection Process Framework. Two types of hospital indicator selection processes were identified. Hospitals deployed most elements found within the 5-P Indicator Selection Process Framework including setting clear aims, having governance structures, considering indicators required by health agencies, and categorizing indicators into strategic themes. Framework elements largely absent included: adopting evidence-based selection criteria; incorporating finance and human resources indicators; considering if indicators measure structures, processes, or outcomes; and engaging a broader set of end users in the selection process. Hospitals have difficulty in balancing how to monitor government-mandated indicators with indicators more relevant to local operations. Hospitals often do not involve frontline managers in indicator selection processes. Not engaging frontline managers in selecting indicators may risk hospitals only choosing government-mandated indicators that are not reflective of frontline operations or valued by those managers accountable for improving unit-level performance.

## Introduction

Given the financial resources dedicated to patient care, governments and private insurers around the world have mandated healthcare organizations to monitor hundreds of indicators to help improve outcomes and enhance accountability [1–6]. An unintended consequence of these well-intended mandates is an overabundance of indicators that can paralyze decision-making and create distrust between providers, government agencies, and insurance companies [1–3, 6–10]. Consequently, several agencies recommend reducing the number of indicators monitored by healthcare organizations [10–14]. However, to identify which indicators should be eliminated, one must understand indicator selection processes.

Current literature focuses on how individual clinical disciplines select indicators. Little research examines the methods that provider agencies, such as hospitals, use to select critical operational indicators to measure organizational performance, while complying with regulatory reporting that may not match operational needs [15]. A recent scoping review resulted in the development of the 5-P Indicator Selection Process Framework which offers an evidence-based structure to design indicator selection processes [15]. As illustrated in Table 1, the framework’s five domains (Purpose, Polity, Prepare, Procedure, and Prove) and 17 elements represent a best practice checklist that helps organization determine why they are selecting indicators; who should govern and select indicators; and how to compile, assess, and validate a final list of indicators [15].

**Table 1.** The 5-P Indicator Selection Process Framework [15].

Domain	Elements	Element description
Purpose	Clarify Aim	Articulate the rationale for conducting an indicator and target selection exercise. By stating the process aim, whether it is to align indicators to an operational process, a strategic plan, a regulatory requirement, or public reporting, the work can be scoped properly.
	Develop Guiding Principles	Establish principles to ensure participants understand the values by which the process is being conducted. Principles may include openness, transparency, scientific soundness, relevance, accountability, scope, and span of control.
	Identify Level of Use	Identify the organizational unit that will use the indicators to ensure relevancy to end users. As an example, indicators used by a Board to monitor quality outcomes may be different from indicators selected by a clinical unit focused on process improvement.
Polity	Build Governance Structures	Identify a structure that will manage indicator and target selection to ensure it is completed. These structures may include a steering committee, a project management team, a data quality advisory group, and an expert panel that will assess potential indicators and targets.
	Recruit Participants	Select and recruit expert panel members. Panels should be diverse and multidisciplinary to ensure equity and a broad view of how indicators and targets will be used. Composition of panels should consider the process aim and level of use when selecting participants.
Prepare	Seek End User Input	Seek input from end users to understand their experiences with the potential indicators under consideration and solicit ideas on the draft criteria they may recommend in evaluating indicators.
	Research Evidence-Based Literature	Identify the range of indicators used in their area or that are required by regulation. A search of literature and evidence-based guidelines and government-mandated indicators will help organizations identify a comprehensive set of indicators to assess.
	Build an Inventory of Potential Indicators	Compile a comprehensive list of indicators with definitions and data sources, so participants understand each indicator to be evaluated. If the process addresses target selection, the nature of the target (e.g. past performance, benchmark, and best practice) should be explained.
	Categorize Potential Indicators into Strategic Themes	Categorize indicators into themes aligned with the organization’s strategy, quadrants of the balanced scorecard, or the Donabedian framework of outcomes, process, and structure. By creating categories, process participants and end users will better understand the linkage an indicator has with the identified purpose.
	Orient and Train Participants	Provide participants with orientation materials on the process aim, definition and purpose of each indicator, potential targets, and methods they will use to recommend indicators and targets.
Procedure	Utilize a Consensus-Building Method	Identify and use a recognized consensus-building method such as the Delphi, modified Delphi, or Normative Group Technique. This is particularly important when indicators are being identified to measure a new strategy compared to a quality improvement project.
	Identify a Facilitator	Select an independent facilitator so as to not bias the process. The facilitator should be a third party or a neutral party from an organization’s performance measurement department.
	Indicator Selection Criteria	Set criteria by which the assessment of indicators will be based. Common criteria include those prescribed by the Appraisal of Indicators through Research and Evaluation tool such as relevance, scientific soundness, feasibility, and validity. Criteria may change based on the aim statement and level of use described in the “Purpose” domain.
	Analytically Assess Indicators	Identify a Likert assessment scale participants will use to evaluate indicators against criteria, and how assessments will be completed, via survey, in person, or both.
	Set Indicator Targets	Assign a target for each indicator. Considerations may include maintaining performance if the current indicators’ result is ahead of a benchmark, attempting to reach a benchmark if performance is behind ideal performance, or making progress toward the benchmark should it be deemed unattainable within the period in which the indicator is being measured.
Prove	Assess Data Quality	Validate the final list of indicators by testing data quality. Processes may wish to defer the setting of specific indicator targets until after this phase to ensure targets are based on valid data trends.
	Validate with End Users	Seek feedback from end users on the relevance the final set of indicators and targets have to their environment and performance requirements, and whether the identified target motivates the end user to implement improvement actions.

## Methods

This study researched the question: “What processes do hospitals in Ontario, Canada use to select performance indicators and how do they align with the 5-P Indicator Selection Process Framework?” The study’s exploratory qualitative design included two data collection methods: semistructured interviews and document analysis [16].

In Ontario, Canada, there are 147 hospitals publicly funded through a single government agency. Fifty-seven operate 48% of the province’s beds and are classified as large community acute care hospitals [17]. Purposeful sampling was used. Four hospitals operating at least 400 beds with annual revenues greater than $400 Million Canadian Dollars were studied [17]. These parameters ensured that hospital cases had comparable services and data reporting requirements. Across four hospitals, 13 executives responsible for reporting on indicators were interviewed with three respondents from Cases A, B, and D and four respondents from Case C. University and all hospital Research Ethics Boards approved the project.

Respondents received a consent form, copy of the 5-P Indicator Selection Process Framework, and interview protocol. Respondents were interviewed about their hospital’s most recent indicator selection process. Interviews were conducted by the first author between June and August 2021, were 45–60 min in length, audio-recorded, and transcribed. Interview data were analyzed using inductive techniques, and responses were aggregated into thematic findings to ensure that contributions were anonymous, protected from social risks, and reflected a common perspective [16]. Document analysis of data presentations and briefing notes were triangulated with interview answers [16]. Data coding was completed by the first author and validated by remaining authors. Member checking was completed by sending aggregated, anonymized summaries, from the first author to respondents individually to validate data [16]. Thus, participants’ identities were known only by the first author.

## Results: case findings

Illustrated in Table 2, two indicator selection processes were identified from analysis of the four cases. Table 3 maps each case process to the framework.

**Table 2.** Ontario hospital indicator selection process types.

No.	Process	Description
1	Renewal of indicators to measure annual operational performance	Annual process to select indicators that will measure yearly goals and objectives. Governed by the senior executive team and corporate data support departments such as business analytics, finance, and quality recommend indicators to the senior team. Senior executive reports indicator results to their board, but the board is not formally involved in indicator selection. Clinical directors and physician leaders are informally consulted but are aware of the indicators being recommended. Process may or may not be documented.
2	Selection of indicators following the completion of a new strategic plan	A formal, structured, and time-limited process aimed at selecting indicators that measure a new strategic plan. Process is governed by the board and senior executive team and involves clinical director and physician leader participation in the selection of indicators and targets. Process is documented and outlines aim, guiding principles, indicator selection criteria, and target setting rationales.

**Table 3.** Case findings compared to the 5-P Indicator Selection Process Framework.

Framework domain/element	Case A	Case B	Case C	Case D
Indicator Selection Process	Process 1	Process 2	Process 2	Process 1
Purpose				
Clarify Aim	To improve quality, accountability, and patient safety and to match provincial funding requirements.	To measure business performance, align organization, enable peer comparison, and meet reporting requirements.	To support adoption of clinical best practices, help run the business more efficiently, and improve accountability.	To select indicators that will improve quality and measuring success against its strategic directions.
Develop Guiding Principles	Does not have a set of guiding principles.	Communicating objectives, assisting timely decisions, leadership engagement, and accountability.	Simplicity (selecting a manageable number of indicators), valuing accuracy over precision, and data availability.	Openness, transparency, alignment, and leadership engagement.
Identify Level of Use	Selects indicators for board and hospital-wide operations.	Selects indicators for board and hospital-wide operations.	Selects indicators for board and hospital-wide operations.	Selects indicators for board and hospital-wide operations.
Polity				
Build Governance Structures	Senior executive is responsible for indicator and target setting and reports to the board.	Board sponsored process with senior executive recommending indicators and targets.	Board sponsored process with senior executive recommending indicators and targets.	Board sponsored process with senior executive recommending indicators and targets.
Recruit Participants	Informally consult clinical directors and physician leaders. Unit managers, patients, and family do not participate in the process.	Process includes executives, clinical directors, and physician leader participation. Unit managers, patients, and family do not participate in the process.	Process includes executives, clinical directors, and physician leader participation. Unit managers, patients, and family do not participate in the process.	Annual process includes executives, clinical directors, and physician leader participation. Unit managers, patients, and family do not participate in the process.
Prepare				
Seek End User Input	Seeks informal input from clinical directors but not managers, patients, or frontline clinicians.	Informally consults on clinical indicators but not finance and HR indicators.	Does not formally consult end users ahead of any indicator or target setting process.	Seeks informal input from clinical directors but not managers, patients, or frontline clinicians.
Research Evidence-Based Literature	Does not research indicator literature but considers indicators measured by peer hospitals and government agencies or national health institutes.	Does not research indicator literature but considers indicators measured by peer hospitals and government agencies or national health institutes.	Does not research indicator literature but considers indicators measured by peer hospitals and government agencies or national health institutes.	Does not research indicator literature but considers indicators measured by peer hospitals and government agencies or national health institutes.
Build an Inventory of Potential indicators	Produces definitions and target rationales for board’s quality improvement plan.	Produces indicator definitions and data sources. Does not include target rationales.	Produces indicator definitions, data sources, and targets. Does not include target rationales.	Produces indicator definitions, data sources, and target justifications when selecting indicators.
Categorize Potential indicators into Strategic Themes	Categorizes indicators into themes that match their strategic plan. Does not consider if an indicator is a process or outcome indicator.	Categorizes indicators into the quadrants of their balanced scorecard. Considers if an indicator is a process or outcome indicator.	Categorizes indicators into the quadrants of their balanced scorecard Does not consider if an indicator is a process or outcome indicator.	Categorizes indicators into the quadrants of their balanced scorecard. Does not consider if an indicator is a process or outcome indicator.
Orient and Train Participants	Does not offer formal training or orientation materials on how to select indicators and targets.	Held training sessions when selecting indicators for a new strategic plan.	Training materials used for selecting indicators for a new strategic plan.	Does not offer formal training or orientation materials on how to select indicators and targets.
Procedure				
Utilize a Consensus-Building Method	Does not use a formal consensus methodology.	Does not use a formal consensus methodology.	Does not use a formal consensus methodology.	Does not use a formal consensus methodology.
Identify a Facilitator	Process facilitated internally by decision support department.	Process facilitated internally by strategy department.	External facilitator used following new strategic plan.	Process facilitated internally by strategy, business analytics, and quality departments.
Establish Indicator Selection Criteria	Data quality, timeliness, funding and public reporting requirements, and clinical relevance.	Data automation, quality, timeliness, usability, funding requirements, and clinical relevance.	Data quality, data availability, gap in performance, and clinical relevance.	Data quality and availability, strategic alignment, benchmarking, and quality improvement.
Analytically Assess indicators	Do not vote on an indicator list. Indicators selected by informal agreement.	Do not vote on an indicator list. Indicators selected by informal agreement.	Do not vote on an indicator list. Indicators selected by informal agreement.	Do not vote on an indicator list. Indicators selected by informal agreement.
Set indicator Targets	Selects targets by first meeting provincial benchmarks. No target setting philosophy on finance or human resource (HR) indicators.	Selects targets by benchmarking against peer performance then incrementally reaching top 25th percentile. Finance and HR indicator targets align to fiscal plan.	If performance is below 50th percentile, target set to 50th percentile. If performance is above 50th percentile, target set to top 25th percentile. If above top 25th percentile, target to maintain performance. Finance and HR indicator targets align to fiscal plan.	Target setting considers own performance, peer performance, and government benchmarks. Selects targets for quality indicators at top 25th percentile. No target setting philosophy on finance and HR indicators.
Prove				
Assess Data Quality	Does not validate any indicators for data quality given many indicators are tested by provincial and national agencies.	Does not validate any indicators for data quality given many indicators are tested by provincial and national agencies.	Does not validate any final list of indicators for data quality given their selection criteria include data quality.	Does not validate government- or agency-mandated indicators but does validate locally driven indicators.
Validate with End Users	No direct validation. Final indicators shared through hospital committees, website, and public data Boards.	No direct validation. Final indicators shared with directors and physician leaders who share indicators across organization.	No direct validation. Final indicators shared with directors and physician leaders who share indicators across organization.	No direct validation. Final indicators shared on public reporting Boards.

### Case A—Process 1

Case A selected indicators at the beginning of its fiscal year. The process this study explored was conducted in 2020. Case A did not document its selection methods. Case A described improving quality, accountability, and funding requirements as process aims. Executives led the process with support from the finance and quality departments. The process did not include a formal consensus methodology. While a draft indicator list was shared with clinical directors and physician leaders, Case A did not involve either group in final indicator selection. Executives set annual targets based on reaching the top 25th percentile performance in their peer group.

Frontline managers did not provide input on the indicators being monitored. Case A—Executive 2 noted this opportunity, “We’ve successfully hardwired a meeting that brings leaders together to go over data, but those metrics are picked by executives. Moving forward we need to seek feedback from clinical units to see if they would change anything.” Case A’s process aligns to only a few domains of the framework (Table 4).

**Table 4.** Case Alignment with 5-P Indicator Selection Process Framework Summary.

Domain	Elements	Case A	Case B	Case C	Case D
Purpose	Clarify Aim	A	A	A	A
	Develop Guiding Principles	NA	A	A	PA
	Identify Level of Use	A	A	A	A
Polity	Build Governance Structures	PA	A	A	PA
	Recruit Participants	NA	PA	PA	PA
Prepare	Seek End User Input	NA	PA	PA	PA
	Research Evidence-Based Literature	A	A	A	A
	Build an Inventory of Potential Indicators	PA	A	A	A
	Categorize Potential Indicators into Strategic Themes	A	A	A	A
	Orient and Train Participants	NA	A	A	NA
Process	Utilize a Consensus-Building Method	NA	NA	NA	NA
	Identify a Facilitator	A	A	A	A
	Establish Indicator Selection Criteria	PA	A	A	A
	Analytically Assess Indicators	NA	NA	NA	NA
	Set Indicator Targets	PA	A	A	A
Prove	Assess Data Quality	NA	NA	NA	PA
	Validate with End Users	PA	NA	NA	NA

Abbreviations: A = Alignment, PA = Partial Alignment, NA = No Alignment.

### Case B—Process 2

Case B completed an indicator selection process following its 2019 strategic plan, which aimed to select indicators that would measure business performance, enable benchmarking, meet reporting guidelines, and ensure organizational alignment. Facilitated by the strategy department, a working group identified initial indicators mandated by government agencies and peers. Directors and physician leaders recommended a final list to executives and the board who cogoverned the process. Approved indicators were shared with frontline managers for information only and not to seek validation. Unique aspects of Case B’s process included having data automation as a selection criterion and that they sought to balance the number of process, outcome, and structural indicators. Case B—Executive 2 shared that one of their goals was to “use our process to get directors, physicians and executives to use the same nomenclature, so we knew how to work together.” In summary, Case B’s process aligns to several elements of the framework (Table 4).

### Case C—Process 2

Case C conducted an indicator selection process following its 2019 strategic plan. The aim of the process was to support improvement, measure business performance, and enhance accountability. Case C—Executive 1 stated, “We’ve put an emphasis on picking indicators that make our leaders accountable for our entire business performance. That means focusing on quality, but also paying attention to finance and human resource metrics.”

Case C’s executives asked clinical directors and physician leaders to draft a list of indicators before they made recommendations to their board. Initial indicators were generated from government and peer scorecards. A formal consensus methodology was not used. Participants were oriented on the process. The final indicator list was not validated by frontline managers. A unique aspect of Case C’s process was its aim to make the process simple by encouraging participants to select a manageable number of indicators. Case C—Executive 2 noted that the process was facilitated by an external consultant, stating “We needed an outside expert to challenge previous biases to measurement.” In summary, Case C’s process aligned with most of the framework (Table 4).

### Case D—Process 1

Case D reviewed indicators at the beginning of each fiscal year. The process this study explored was conducted in 2020. Case D documented the process, which aimed to measure its strategic performance. The process was governed by its executives and board with openness and transparency as guiding principles. Indicator selection criteria included data quality, data availability, and ability to benchmark. Case D was in the process of reviewing these criteria. As Case D—Executive 3 shared,

We’ve made indicator selection too simple by only accepting administrative outcome data. Patient conditions are very complex. If I had to re-select the indicators we monitor, I would focus on clinical process indicators like how many specimens are lost, or number of cancers misdiagnosed, or outcome indicators that address unnecessary deaths and then use risk adjustments so clinicians can make direct practice changes.

Unique aspects of Case D’s process included the documentation of indicator selection criteria, definitions, and target justifications. In summary, Case D’s process matched some elements of the framework (Table 4).

## Results: multicase analysis

The following section describes the common alignment and deficiencies case processes had compared to the framework.

### Domain 1: Purpose

All hospitals articulated the aim of their processes, with Cases B, C, and D documenting them. Aim statements included selecting indicators that measured business performance, supported quality improvement, and met reporting requirements. Case A did not identify guiding principles related to their process, whereas Case B articulated principles, but they were not documented. Cases C and D documented guiding principles. Openness, transparency, and accountability were common guiding principles.

### Domain 2: Polity

Executives led their respective processes. Cases B, C, and D required board approval of final indicator lists, while Case A did not report having to do so. Process participants included executives, directors, and physician leaders. Absent from all governance structures were end users such as frontline managers.

### Domain 3: Prepare

Government-mandated indicators were prioritized given they are often essential to obtain funding. All hospitals noted that the maintenance of indicator lists and definitions was inconsistent and an area for improvement. Cases B and C oriented participants on indicator selection given their processes followed new strategic plans. Cases A and D did not orientate participants to their annual processes.

### Domain 4: Procedure

No case used a consensus-building methodology such as a Delphi process [18], arguing that the absence of a consensus method was influenced by their culture. Case B—Executive 1 stated, “We would never put a list of indicators in front of our leaders to vote on. Given we need to work together every day, it is just not in our culture. If there is a disagreement, that would be up to executives to decide.”

Cases A, B, and D had internal staff facilitate their processes, while Case C used external facilitation. Selection criteria did not vary significantly across cases. Data quality, availability, and comparability were common criteria. All hospitals considered an indicator if it was required to obtain funding. Case B considered whether an indicator was a process, structure, or outcome indicator.

In setting targets, hospitals relied on benchmarks published by government and policy agencies. Cases B and C’s target setting philosophies were based on achieving the top 25th percentile of their peer group. In setting targets, Case D analyzed annual performance and then considered peer and government benchmarks. Target setting philosophies were evident for quality indicators, but not financial or human resources indicators.

### Domain 5: Prove

No case quantitatively validated their indicators for data quality, reasoning that they generally selected government-mandated indicators. Hospitals did not seek input from frontline managers or patients on the face validity of indicators, opting to share a list of indicators for information only. Case B—Executive 3 explained, “We present our indicators to our patient council. But it is more of a report to them than active engagement.”

## Discussion

### Statement of principal findings

This study identified two types of indicator selection processes: the renewal of indicators to measure annual operational performance and selection of indicators following the completion of a new strategic plan (Table 2). Table 4 provides a comparison of case processes and how they align with the 5-P Indicator Selection Process Framework. No process mirrored the framework completely. Cases A and D, who completed processes as part of their annual planning cycle, were less aligned with the framework, whereas Cases B and C, who completed processes following strategic plans, were more aligned. Common findings are categorized into three themes: the structure and mechanics of indicator selection processes, the engagement of end users, and documentation.

Structure and mechanics of indicator selection processes is the first area of findings in which gaps existed. These gaps are found in the following framework elements: Clarify Aim, Identify a Facilitator, Indicator Selection Criteria, Set Indicator Targets, and Assess Data.

All hospitals confused setting process aims and guiding principles. The aim of a process may be to select indicators that measure business performance or quality, whereas the principles guiding the process may be openness, transparency, and accountability. Setting clear aims and guiding principles allows participants to understand process’s goals and how they may contribute [19]. Hospitals generally relied on internal staff to facilitate the process. Internal facilitation may bias processes, whereas external facilitators can manage discussions that might otherwise be difficult [20]. Given indicator selection processes are linked to measuring goals and accountabilities, processes would be improved with external facilitation.

Hospitals focused on selecting quality and patient experience indicators. However, hospitals are not only medical agencies, but business units with economic impact. Hospitals would benefit from having finance and human resources indicators as part of their selection processes. The Donabedian framework advocates that quality is measured using three types of indicators: structural, process, and outcome [21]. Only Case B used the Donabedian framework as a criterion for selecting indicators. Hospitals should consider the Donabedian framework when selecting indicators to explore the cause and effect relationships that influence quality.

All four cases stated that government funding requirements were criteria they strongly considered. This led hospitals to question how to balance monitoring indicators that measure local needs while also monitoring mandated indicators that may be less relevant. This finding confirms earlier literature that found government-mandated indicators that are not regularly renewed are less relevant to operations and create confusion [2, 3, 5, 6, 22]. To streamline indicator selection, hospitals and funding agencies should align criteria to instruments such as the Appraisal of Indicators through Research and Evaluation tool [23]. Of course, not all hospitals operate in a single-payer system; we note that in multipayer systems, as in the USA, payers may have different priorities, which may add further complexity to indicator selection.

Study participants identified that target setting needed improvement. They attributed this need to their reliance on government-mandated metrics, overuse of benchmarks, and an inability to attribute numerical gains to improvement activities.

No case quantitatively validated their list of indicators for data quality. Hospitals reasoned that they did not need to complete this validation given most indicators they chose were from funding agencies. This is a false assumption. While an indicator’s technical formula may be validated by an external agency, data generated from local information systems may be of different quality. Confidence in hospital processes may be improved if they validate local data quality.

The most glaring deficiency of the processes studied was in the engagement of end users in indicator selection. This gap is associated with the following framework elements: Recruit Participants, Seek End User Input, Orient and Train Participants, Validate with End Users, and Utilize a Consensus-Building Method [15].

Hospital board involvement seemed perfunctory. Boards appeared to only approve a recommended list, rather than actively participating in indicator selection. While cases oriented participants on measurement if a process followed a strategic plan (Process 2), hospitals did not orient participants on annual processes (Process 1). These are important reflection points. Involving boards in indicator selection helps improve outcomes [24, 25]. Processes that have broader participation have a deeper understanding of frontline operations [15]. Participants who understand why a project is undertaken are more likely to positively contribute to the initiative [26, 27]. Excluding individuals who are accountable for service delivery can lead to selecting indicators that do not match operational realities [6, 9, 28].

All cases stated that time demands prohibited them from using consensus-building methodologies. Delphi techniques allow transparent debate while reducing bias and groupthink [18]. Given all cases desired to build collaborative cultures, Delphi techniques should be seen as helpful and not a hindrance.

Documentation generally took the form of briefing notes and presentations. To ensure indicator selection processes are defined and understood, hospitals should document their approaches in the form of policies and procedures. Utilizing the framework as a basis for documentation will ensure that key elements of the process are considered.

### Strengths and limitations

This paper represents the first real-world assessment of indicator selection processes using the 5-P Indicator Selection Process Framework. A qualitative multiple case study approach enabled a systematic exploration of participant experiences across different hospitals. However, this study also has several limitations. Its focus on large community acute care hospitals in Ontario, Canada, may limit the generalizability of findings to other hospital types. This study examined hospitals funded by a single public agency; many others are subject to multiple public and private reimbursement agencies. The study was completed during the corona virus disease (COVID-19) pandemic: this may have impacted participant recall given hospitals may have stopped reviewing indicators during that period. This study researched executive perceptions of indicator selection and not those who participated in the described processes, who may have had different perceptions. While we were fortunate to gain access to executives, the sample (*n* = 13) is relatively small. Finally, respondents received an advance copy of the framework that may have influenced their responses.

### Implications for policy, practice, and research

This study identified two types of hospital indicator selection processes that are heavily influenced by indicators mandated by regulatory and funding agencies. For policymakers, this study may inform how they might standardize the methods by which indicators are selected and retired. For practitioners, this study emphasizes the need to include frontline managers in indicator selection processes, so they can understand how they can contribute to improving indicator performance. An objective of the framework is to guide better collaboration and decision-making. Future research may therefore study how the framework informs proactive planning of indicator selection and how indicators are used for operational decision-making.

### Interpretation within the context of the wider literature

There is much research on consensus-building exercises to select indicators for specific clinical areas compared to studying how hospital organizations select indicators [15]. This study helps address that gap by being the first real-world assessment of four hospital indicator selection processes using the 5-P framework.

## Conclusion

Reducing the number of indicators required by both regulatory and payment bodies is a major challenge for healthcare systems that will take time to achieve. In the meantime, hospital executives must help their organizations select an appropriate number of critical indicators to help their frontline managers achieve local strategic and operational goals. This study identified two indicator selection processes used by hospitals: annual renewal of indicators to measure operational performance and selection of indicators following the completion of a new strategic plan. Gaps in these processes compared to the 5-P Indicator Selection Process Framework included the lack of guiding principles; consensus-building methodologies; evidence-based selection criteria; business-based indicators; balance between structural, process, and outcome indicators; and validation. The most glaring gap was the lack of engagement of frontline unit managers in these processes. Frontline managers should be active participants in indicator selection given their accountability for implementing changes designed to improve outcomes.

## Data Availability

The data underlying this qualitative, multiple case study cannot be shared publicly due to the privacy of the individuals that participated in the study. Aggregated and anonymized data will be shared on reasonable request to the corresponding author.
